# Predictive value and ranking of writhing and fidgety movements for cerebral palsy: A meta-analysis based on the Superiority Index

**DOI:** 10.1097/MD.0000000000043813

**Published:** 2025-08-15

**Authors:** Taotao Wang, Bo Zheng, Hong Yang, Hongyan Liu, Yun Zhou, Zhijie Wen, Yuanfei Ye, Long Guo

**Affiliations:** a Xi’an Daxing Hospital, Xi’an, China; b Yuhuan People’s Hospital, Taizhou, China; c Children’s Hospital of Fudan University, Shanghai, China; d Changde Maternal and Child Health Hospital, Changde, China; e Wu’an First People’s Hospital, Wu’an, China; f Xuancheng People’s Hospital, Xuancheng, China.

**Keywords:** cerebral palsy, fidgety, general movements, meta-analysis, sensitivity, specificity, Superiority Index, writhing

## Abstract

**Background:**

This meta-analysis assessed the diagnostic value of writhing and fidgety general movements (GMs) for predicting cerebral palsy.

**Methods:**

We searched PubMed, EMBASE, Cochrane Library, CINAHL, and Web of Science for cohort or case-control studies evaluating GMs. We calculated the superiority index (S), sensitivity, specificity, likelihood ratios, diagnostic odds ratio (DOR), and area under the curve (AUC) using STATA.

**Results:**

Eight studies were included. For writhing movements, S = 1, sensitivity = 0.99, specificity = 0.69, AUC = 0.91, and DOR = 166. For fidgety movements, S = 3, sensitivity = 0.95, specificity = 0.87, AUC = 0.97, and DOR = 144. Subgroup analyses by study design, risk population, and sample size consistently showed better predictive performance of fidgety movements. For example, in high-risk children, AUC was 0.98 for both periods, but DOR was higher for fidgety (169 vs 302 for writhing). Across subgroups, the Superiority Index of fidgety remained stable at 3.

**Conclusion:**

Fidgety GMs have significantly greater predictive value for cerebral palsy than writhing GMs. This advantage remains consistent across study types, populations, and sample sizes.

## 1. Introduction

Cerebral palsy (CP) is a group of permanent movement and posture disorders attributed to nonprogressive disturbances in the developing fetal or infant brain. It is the most common cause of physical disability in early childhood, with a global prevalence of approximately 2 to 3 per 1000 live births.^[1]^ Early detection is crucial to initiate timely intervention and improve neurodevelopmental outcomes.

General Movements (GMs), first described by Prechtl, are spontaneous movements that appear early in fetal life and reflect the integrity of the central nervous system.^[2]^ The assessment of GMs has emerged as one of the most reliable tools for the early prediction of CP in high-risk infants.^[3]^ Two key developmental stages of GMs are particularly relevant: the writhing period (from birth to approximately 6 to 9 weeks post-term) and the fidgety period (from 9 to 20 weeks post-term).

During the writhing period, GMs are characterized by slow, large-amplitude, and complex movements involving the whole body. Abnormal patterns during this stage, such as poor repertoire or cramped-synchronized movements, may suggest neurological dysfunction but lack specificity.^[4]^ In contrast, fidgety movements, observed in the fidgety period, are small movements of moderate speed and variable acceleration occurring irregularly all over the body. The absence of fidgety movements has been strongly associated with the later diagnosis of CP and is considered a highly specific and sensitive predictor.^[5]^

Given that different GMs patterns may vary in their predictive value depending on the developmental stage, it is clinically important to compare their diagnostic accuracy directly. Traditional pairwise meta-analyses cannot address this adequately when multiple diagnostic options are available.

While ranking of tests using rank probabilities and rank grams is an attractive feature, it is still a challenge to rank competing diagnostic tests especially when a test does not outperform the others on both sensitivity and specificity. Consider the diagnostic odds ratio (DOR) cannot distinguish between tests with high sensitivity but low specificity or vice-versa. Alternatively, the superiority of a diagnostic test could be quantified using a superiority index expressed as S_k_ = (2a_k_ + c_k_)/ (2b_k_ + c_k_), where a_k_ is the number of tests to which test k is superior (higher sensitivity and specificity), b_k_ is the number of tests to which test k is inferior (lower sensitivity and specificity) and c_k_ the number of tests with equal performance as test k (equal sensitivity and specificity). S ranges from 0 to 1 with S tending to 1 and S tending to 0 as the number of tests to which test k is superior and inferior increases, respectively, and S tending to 1 the more the tests are equal. Since the number of tests not comparable to test k does not enter the calculation of S, the index for different tests may be based on different sets of tests.^[6]^

This study aims to systematically compare the diagnostic value of GMs assessment during the writhing and fidgety periods for CP using a Bayesian multiple-treatment comparison model and rank them based on S.

## 2. Methods

### 2.1. Overview

Our study protocol, which has been registered in the International Prospective Register of Systematic Reviews (CRD42024560789), and the Cochrane handbook and diagnostic accuracy study reviews were used to guide the analysis.^[7,8]^ The study followed a prior established protocol.

### 2.2. Eligibility criteria

Type of studies. We had planned to include both case-control studies and cohort studies as well as prospective and retrospective studies, as long as they could provide sufficient data concerning both the sensitivity and specificity of the writhing period and fidgety period for CP.^[9,10]^ There is no language limitation for searching. Studies that only covered sensitivity or specificity were excluded.

#### 2.2.1. *Literature search strategy*:

In the electronic search, we systematically searched PubMed, CINAL, EMBASE, the Cochrane Library, and Web of Science on March 16, 2025. Two investigators independently screened the candidate articles by checking the title and abstract after uploading the citation list into ENDNOTE 20.4 (Bld.18004). After independent screening, articles that were still regarded as candidates by at least 1 investigator were then independently scrutinized through full-text reading. The final inclusion was decided after resolving discrepancies between the investigators.

### 2.3. Search strategy

The following descriptors were included: “GMs” (Title/Abstract) and “CP” (Title/Abstract) and “sensitivity” (Title/Abstract), simplified. As the aim is to compare the diagnosis of writhing period and fidgety period, so we would include the article did the test for both periods, exclude the article which just do 1 period GMs. The full descriptor is on this site. The bibliographies of the included studies and recent review articles were also searched to identify additional studies.

### 2.4. Study selection and data extraction

Data were extracted by 2 reviewers independently and then cross-checked. The following data were extracted: baseline study characteristics – primary author, title of publication, year of publication, geographical location of patient population, and study design; basic information like sex, weight, and gestation week, and the proportion of TP, FP, TN, and FN for each diagnostic test.

Study quality was determined using the Quality Assessment of Diagnostic Accuracy Studies 2 (QUADAS-2) tool.^[11–13]^ We used the standard 4 QUADAS-2 domains: patient selection, index test, reference standard, and flow/timing. The patient population was defined as the infants who had the risk of CP, and the index test was defined as writhing and fidgety period of GMs. The reference test was defined as writhing period and fidgety period. The flow/timing domain was defined as all included infants at risk of CP underwent both index tests (GMs assessments during the writhing and/or fidgety periods). Each study was assigned an overall judgment of “low,” “high,” or “unclear” risk by both authors, and all discrepancies were resolved by consensus. For sensitivity analysis, a study with at least 1 domain scored as high risk or high applicability concern was regarded as a high-risk study.

### 2.5. Statistical analysis

Review Manager 5.3,^[12]^ Stata 17, and R were used for statistical analysis. Sensitivity and specificity were calculated for each available comparison. The main outcomes were S, the diagnostic test accuracy evaluated by the following statistics: DOR, area under the curve (AUC), and summary estimates of the sensitivity (SEN), specificity (SPE), positive likelihood ratio (PLR), and negative likelihood ratio (NLR).

We used S for the ranking of diagnosis accuracy of writhing and fidgety periods for CP (The R commands are provided in Supplementary File, Supplemental Digital Content, https://links.lww.com/MD/P634). The S, a ranking measure derived from a Bayesian network meta-analysis, was calculated using a model implemented in Stan via the Rstan package in R. The model estimates the probabilities of true positives and true negatives across studies for each diagnostic strategy. Based on these, the S for each test was computed using pairwise comparisons of sensitivity and specificity, reflecting the probability that a test outperforms others in both dimensions.

We used both the hierarchical summary receiver operating characteristics model and bivariate model. Heterogeneity, which is the degree of inconsistency among studies, was assessed using the *I*^2^ statistic: 0% to 40% represents not important heterogeneity, 30% to 60% represents moderate heterogeneity, 50% to 90% represents substantial heterogeneity, 75% to 100% indicates considerable heterogeneity. To determine overall accuracy, we calculated the DOR and AUC values. DOR is a measure of the effectiveness of a diagnostic test, wherein DOR = 1 means no diagnostic value, DOR > 1 means that a test positive suggests disease positivity, and DOR < 1 means that a test negative suggests disease positivity. We obtained a paired forest plot, hierarchical summary receiver operating characteristic curve, and summary estimates of sensitivity and specificity using the bivariate model. PLR and NLR were calculated from summary estimates of sensitivity and specificity. AUC, PLR, and NLR were interpreted according to the 4-grade criteria.

The AUC indicates how accurate a test is: an AUC in the ranges of < 0.75, 0.75 to 0.92, 0.93 to 0.96, and > 0.97 meant “not accurate good,” “very good” and “excellent,” respectively. PLR defined by “sensitivity/ (1-specifificity)” and NLR defined by “(1 -sensitivity)/specificity” represent how the test results change the probability of a disease. PLR values in the range of < 2, 2 to 5, 5 to 10 and > 10 were recognized as showing a “not meaningful,” “small,” “moderate” and “large” increase in probability, respectively. NLR in the range of >0.5, 0.2 to 0.5, 0.1 to 0.2, and < 0.1 represented a “not meaningful,” “small,” “moderate” and “large” decrease of probability, respectively.

### 2.6. Subgroup and sensitivity analyses

In subgroup analysis, we classified the studies into 3 subgroups: 1 based on whether the article is prospective, 1 based on whether the article focuses on diagnosing high-risk children, and 1 based on sample size, and excluded the studies with fewer than 100 participants. In the sensitivity analysis, we sequentially excluded individual studies and performed Bayesian network meta-analysis to obtain the corresponding sensitivity S and DORs (DOR). We then observed which study caused the largest variation in these 2 values.

### 2.7. Publication bias

Publication bias was assessed graphically by Funnel plots and the Deeks’ Funnel plot asymmetry test, *P* < .5 was defined as a publication bias positive.

## 3. Results

### 3.1. Study search results and characteristics

**Figure 1.**
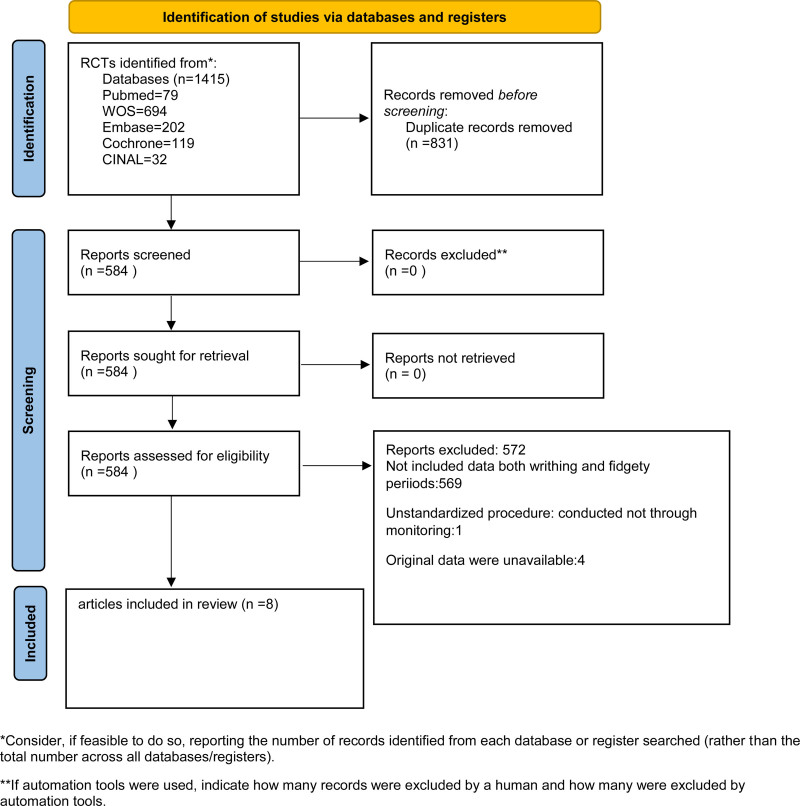
The preferred reporting items in systematic reviews and meta-analyses flow chart for study search.

**Figure 2.**
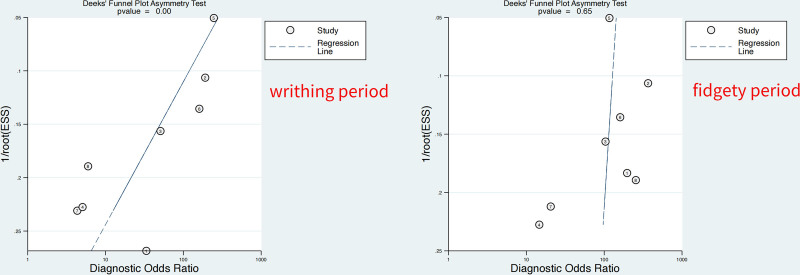
Publication bias of the meta-analysis.

A total of 1625 citations were identified in the initial database search. Based on the abstract review, 17 studies were selected for full-length manuscript evaluation. After applying the inclusion and exclusion criteria, 12 citations were included. Four studies were excluded due to the inability to extract relevant data, and the remaining 8 studies were included in the meta-analysis.^[14–21]^ Figure 1 depicts the study flow. The Deeks’ Funnel Plot Asymmetry Test *P* = .00 for writhing period and 0.65 for fidgety period, so there is a publication bias for the research of writhing period, but no publication bias for the research of fidgety period, as shown in Figure 2.

The study characteristics are summarized in Table 1. Of the studies that were published from 2007 to 2024, 5 studies were prospective,^[16,17,19–21]^ 1 study was retrospective, and 2 studies were unclear.^[14,15]^ Two articles were from Italy,^[14,22]^ 1 from Australia,^[18]^ 2 from Serbia,^[16,19]^ 1 from the Netherlands,^[17]^ 1 from China,^[20]^ and 1 from Kazakhstan.^[21]^

**Table 1:** Patient and study characteristics.

Study ID	Year	Country	Study type	Patient profile	No. randomised	Gestation week (mean ± SD)	Birth weight (g)	CP	Writhing period		Fidgety period		Male (%)
									CP	others	CP	others	
A. Guzzetta	2007	Italy	Unclear	Term	12	39.6 ± 1.5	3471.1 ± 401.1	Yes	3 (28)	0 (28)	7 (112)	0 (112)	Unclear
				Preterm	103	32.2 ± 2.8	1776.3 ± 563.9	No	4 (28)	21 (28)	7 (112)	98 (112)	
C. Brogna	2013	Italy	Unclear	Preterm	574	Unclear	2299 ± 451	Yes	22	0	22	0	Unclear
								No	105	447	60	492	
L. Dimitrijević	2016	Serbia	Prospective	Preterm	79	unclear	unclear	Yes	11	0	11	0	51.9
								No	21	47	12	56	
van Iersel	2016	Netherlands	Prospective	Term	145	40 ± 5.19	3454 ± 601	Yes	3	2	3	2	59
								No	32	108	13	127	
C. Morgan	2019	Australia	Retrospecitve	High-Risk	441	Unclear	Unclear	Yes	146	1	140	7	Unclear
								No	109	184	43	251	
D. Zlatanovic	2022	Serbia	Prospective	Preterm	160	Unclear	Unclear	Yes	14	0	14	0	Unclear
								No	22	124	22	124	
H. Wang	2023	China	Prospective	High-risk(NRDS)	80	Unclear	Unclear	Yes	4	1	5	0	56.25
								No	36	39	26	49	
Z. Zhussupova	2024	Kazakhstan	Prospective	High-risk (HIE)	31	Unclear	Unclear	Yes	9	0	8	1	Unclear
								No	17	5	0	22	

CP = cerebral palsy; NRDS = neonatal respiratory distress syndrome; HIE = hypoxic-ischemic encephalopathy.

One of the articles included low-risk infants,^[17]^ while the remaining 7 focused on high-risk infants, such as those with prematurity, neonatal respiratory distress syndrome, or hypoxic-ischemic encephalopathy. Three of the articles had a sample size of fewer than 100 participants.^[16,20,21]^

### 3.2. Risk of biases assessment

**Figure 3.**
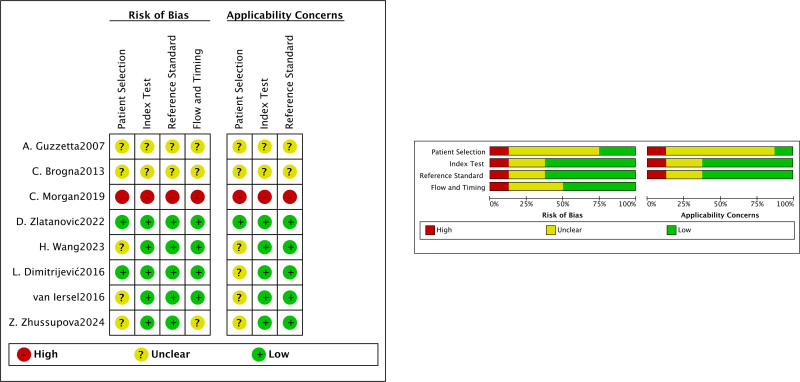
Risk of bias and applicability concerns graph and summary.

The visual representation of bias assessment is shown as a risk of bias (Fig. 3). The assessment showed low to moderate results of bias, except for 1 study, which showed a high result of bias.^[18]^

### 3.3. Meta-analysis of all the studies

**Figure 4.**
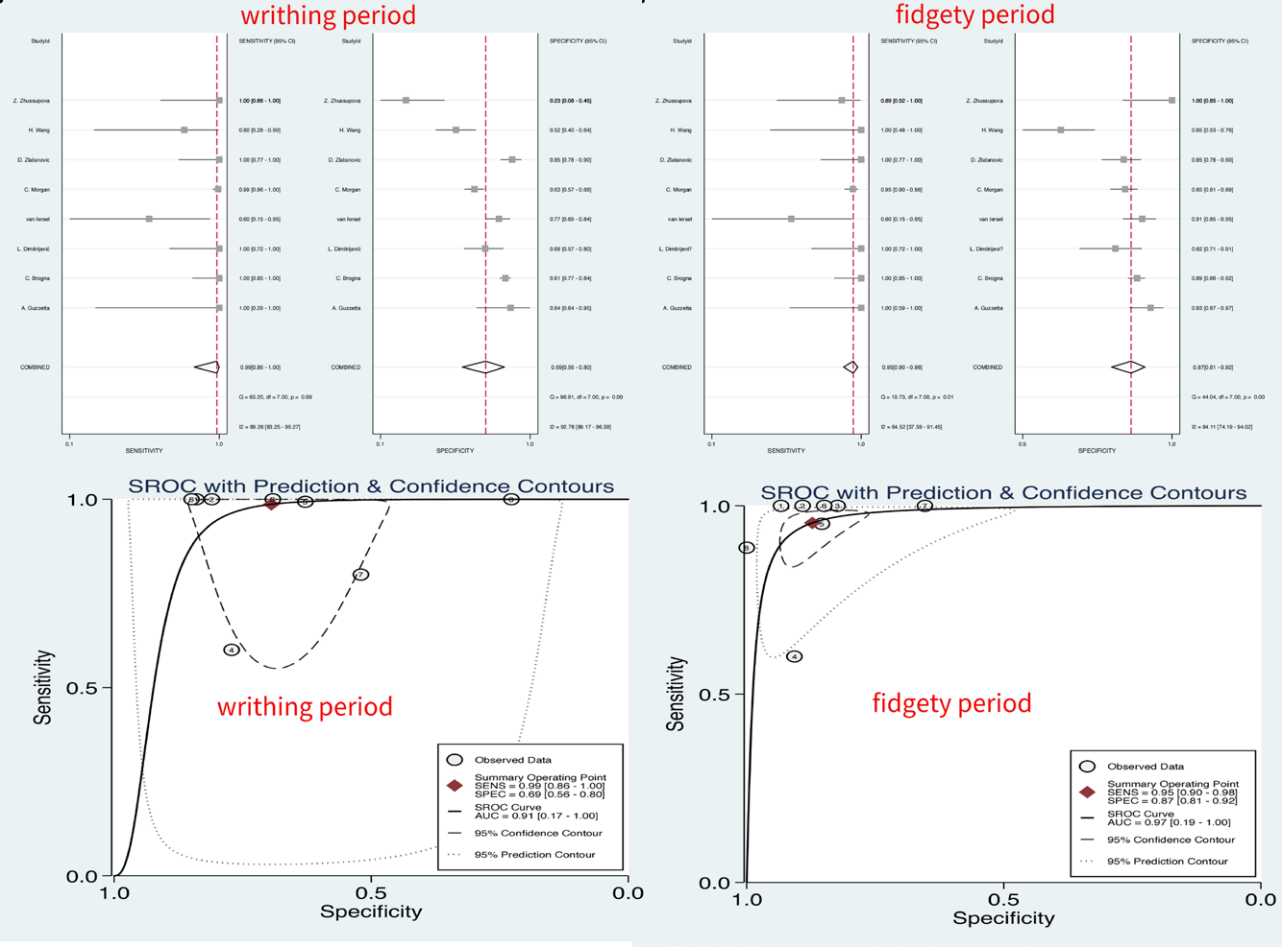
Results of diagnostic meta-analysis, including the pooled sensitivity, specificity, and AUC. AUC = area under the curve.

Used data from all 8 studies, consisting of 220 CP children and 1405 CP-negative children. For writhing period, the summary estimates of S, sensitivity specificity PLR, NLR, DOR, and AUC were 1, 0.99 (0.86, 1.00), 0.69 (0.56, 0.80), 3.2 (2.1, 4.9), 0.02 (0.00, 0.23), 166 (12, 2214), and 0.91 (0.17–1.00) respectively. For fidgety period, the summary estimates of S, sensitivity specificity PLR, NLR, DOR, and AUC were 3, 0.95 (0.90, 0.98), 0.87 (0.81, 0.92), 7.5 (5.1, 10.9), 0.05 (0.02, 0.11), 144 (71, 292), and 0.97 (0.19, 1.00) respectively. Which were shown in Figure 4. I2 were 89.26% for writhing period and 92.78% for fidgety period, which both indicating considerable heterogeneity, and subgroups were considered to reduce the heterogeneity. The S was 1 for writhing period, and 3 for fidgety period.

### 3.4. Subgroup and sensitivity analyses

**Figure 5.**
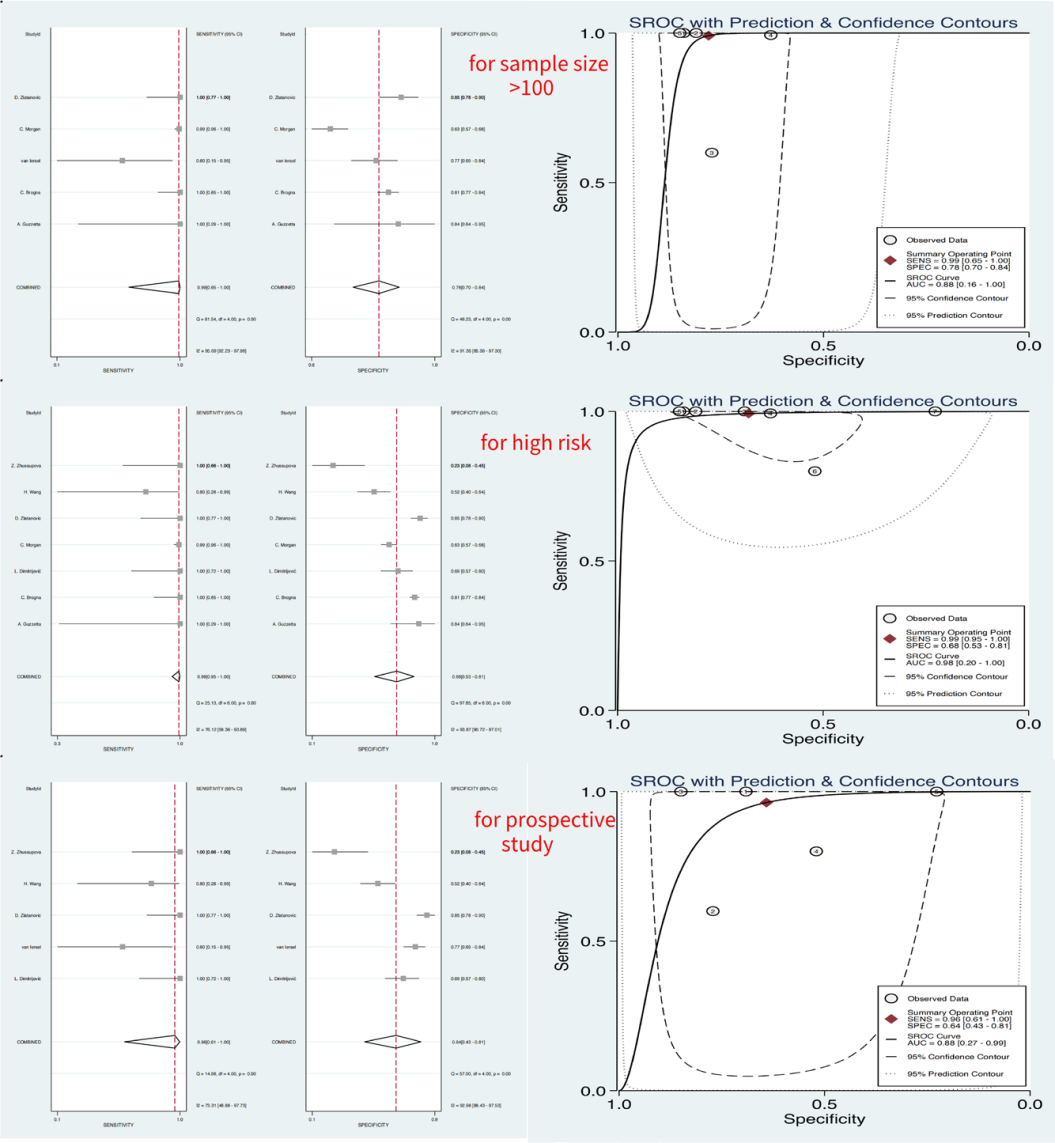
Results of diagnostic meta-analysis for subgroup of writhing period, including the pooled sensitivity, specificity, and AUC. AUC = area under the curve.

**Figure 6.**
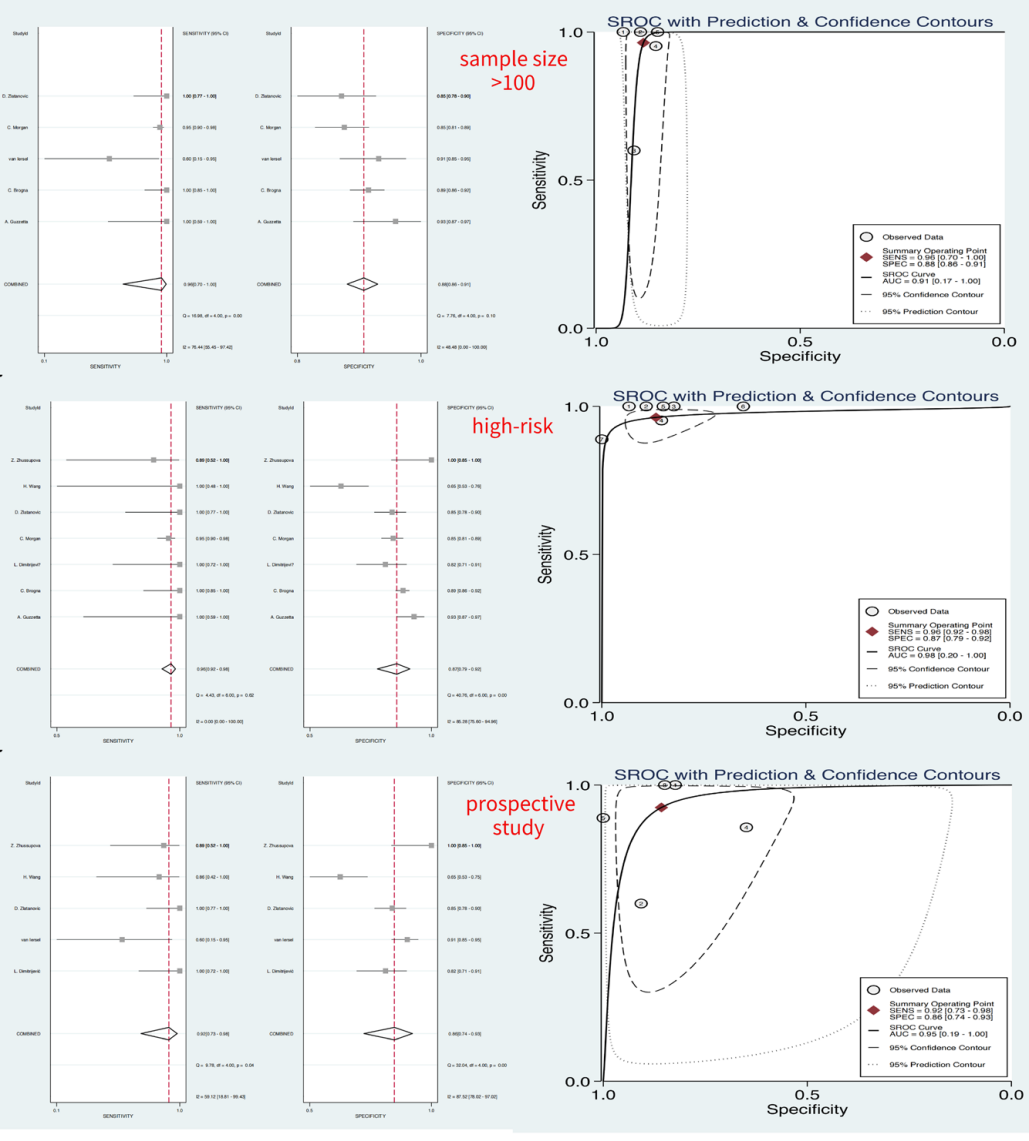
Results of diagnostic meta-analysis for subgroup of fidgety period, including the pooled sensitivity, specificity, and AUC. AUC = area under the curve.

The results of the subgroup analyses based on prospective studies, are shown in Table 2. The S, AUC, and DOR of writhing periods were 1, 0.88 (0.27, 0.99), and 47 (3, 800), as shown in Table 2 and Figure 5. And the S, AUC, and DOR of fidgety periods were 3, 0.95 (0.19, 1.0), and 73 (15, 349), as shown in Table 2, Figures 5 and 6.

**Table 2:** Patient and study characteristics of eligible studies assessing writhing period and fidgety period for cerebral palsy.

	Total		Prospective		High-risk		Sample size > 100	
	Writhing	Fidgety	Writhing	Fidgety	Writhing	Fidgety	Writhing	Fidgety
S value	1	3	1	3	1	3	1	3
DOR	166 (12–2214)	144 (71–292)	47 (3–800)	73 (15–349)	302 (31–2966)	169 (76–372)	402 (6–26369)	202 (18–2260)
AUC	0.91 (0.17–1.00)	0.97 (0.19–1.00)	0.88 (0.27–0.99)	0.95 (0.19–1.0)	0.98 (0.2–1.0)	0.98 (0.2–1.0)	0.88 (0.16–1.0)	0.91 (0.17–1.00)
SEN	0.99 (0.86–1.00)	0.95 (0.90–0.98)	0.96 (0.61–1.00)	0.92 (0.73–0.98)	0.99 (0.95–1.00)	0.96 (0.92–0.98)	0.99 (0.65–1.00)	0.96 (0.70–1.00)
SPE	0.69 (0.56–0.80)	0.87 (0.81–0.92)	0.64 (0.43–0.81)	0.86 (0.74–0.93)	0.68 (0.53–0.81)	0.87 (0.79–0.92)	0.78 (0.70–0.84)	0.88 (0.86–0.91)
PLR	3.2 (2.1–4.9)	7.5 (5.1–10.9)	2.7 (1.5–4.7)	6.5 (3.5–12.2)	3.1 (2.0–4.9)	7.2 (4.6–11.4)	4.5 (3.2–6.3)	8.3 (6.8–10.2)
NLR	0.02 (0.00–0.23)	0.05 (0.02–0.11)	0.06 (0.00–0.84)	0.09 (0.02–0.35)	0.01 (0.00–0.08)	0.04 (0.02–0.09)	0.01 (0.00–0.68)	0.04 (0.00–0.43)

NLR = negative likelihood ratios, PLR = positive likelihood ratios, SEN = sensitivity, SPE = specificity, DOR = diagnostic odds ratios, AUC = area under the curve.

The results of the subgroup analyses based on high-risk children, are shown in Table 2. The S, AUC, and DOR of writhing periods were 1, 0.98 (0.20, 1.0), and 302 (31, 2966), and the S, AUC, and DOR of fidgety periods were 3, 0.98 (0.20, 1.00), and 169 (76, 372), both shown in Table 2, Figures 5 and 6.

The results of the subgroup analyses based on studies with sample sizes exceeding 100, are shown in Table 2. The S, AUC, and DOR of writhing periods were 1, 0.88 (0.16, 1.0), and 402 (6, 26369), And the S, AUC, and DOR of fidgety periods were 3, 0.91 (0.17, 1.00), and 202 (18, 2260), both shown in Table 2, Figures 5 and 6.

In the leave-one-out sensitivity analysis, we identified 1 study as the main source of heterogeneity both for writhing period analysis and fidgety period analysis.^[17]^ This study focused on the assessment of GMs in low-risk infants, which differed from the populations targeted in the other studies. It is therefore likely to be the primary contributor to heterogeneity. Sensitivity and specificity estimate across the 2 time periods showed no substantial changes in the sensitivity analysis, as shown in Table 3.

**Table 3:** The leave-one-out sensitivity analysis.

	I2 (P)		SEN		SPE		PLR		NLR		DOR	
Excluded study	W	F	W	F	W	F	W	F	W	F	W	F
Guzzetta	66 (0.026)	54 (0.057)	0.99 (0.85–1.00)	0.96 (0.87–0.99)	0.67 (0.52–0.79)	0.86 (0.80–0.91)	3.0 (2.0–4.6)	6.9 (4.8–9.8)	0.02 (0.00–0.25)	0.05 (0.01–0.15)	135 (11–1703)	148 (54–408)
Brogna	69 (0.002)	59 (0.043)	0.98 (0.83–1.00)	0.95 (0.88–0.98)	0.67 (0.52–0.80)	0.87 (0.79–0.92)	3.0 (1.9–4.6)	7.2 (4.7–11.0)	0.03 (0.00–0.28)	0.06 (0.02–0.14)	99 (10–994)	124 (59–263)
Dimitrijević	67 (0.024)	41 (0.092)	0.98 (0.84–1.00)	0.95 (0.89–0.98)	0.69 (0.53–0.82)	0.88 (0.81–0.93)	3.2 (2.0–5.2)	7.9 (5.0–12.3)	0.02 (0.00–0.27)	0.06 (0.03–0.12)	129 (10–1634)	137 (69–272)
van Iersel	0 (0.495)	0 (0.273)	0.99 (0.95–1.00)	0.96 (0.92–0.98)	0.68 (0.53–0.81)	0.87 (0.79–0.92)	3.1 (2.0–4.9)	7.2 (4.6–11.4)	0.01 (0.00–0.08)	0.04 (0.02–0.09)	302 (31–2966)	169 (76–372)
Morgan	49 (0.069)	68 (0.021)	0.99 (0.66–1.00)	0.99 (0.56–1.00)	0.70 (0.55–0.82)	0.88 (0.81–0.92)	3.3 (2.0–5.4)	8.0 (5.1–12.7)	0.02 (0.00–0.70)	0.01 (0.00–0.83)	160 (4–6189)	873 (11–71608)
Zlatanovic	68 (0.022)	59 (0.044)	0.98 (0.84–1.00)	0.95 (0.87–0.98)	0.66 (0.52–0.78)	0.87 (0.81–0.92)	2.9 (2.0–4.4)	7.6 (5.0–11.5)	0.03 (0.00–0.27)	0.06 (0.02–0.15)	105 (10–1105)	126 (60–262)
Wang	67 (0.025)	0 (0.37)	0.99 (0.84–1.00)	0.95 (0.89–0.98)	0.72 (0.57–0.83)	0.88 (0.85–0.91)	3.5 (2.2–5.5)	8.2 (6.6–10.3)	0.01 (0.00–0.26)	0.06 (0.03–0.13)	372 (13–10348)	137 (65–287)
Zhussupova	64 (0.03)	15 (0.155)	0.99 (0.80–1.00)	0.98 (0.70–1.00)	0.74 (0.65–0.81)	0.86 (0.80–0.90)	3.8 (2.7–5.2)	7.0 (4.9–9.9)	0.02 (0.00–0.33)	0.02 (0.00–0.44)	229 (10–5216)	288 (16–5142)

DOR = diagnostic odds ratios, NLR = negative likelihood ratios, PLR = positive likelihood ratios, SEN = sensitivity, SPE = specificity.

## 4. Discussion

This meta-analysis provides comprehensive evidence supporting the predictive value of GMs assessment for CP, with a specific focus on the writhing and fidgety periods. Our findings demonstrate that while both periods contribute to the early identification of CP, the fidgety period yields a higher Superiority Index (S), indicating a more robust discriminatory performance in distinguishing infants at risk.

The superiority of the fidgety period may be attributed to its neurodevelopmental underpinnings. The absence of fidgety movements has been consistently associated with adverse motor outcomes, particularly spastic CP.^[2]^ Compared to the writhing period, which may reflect more transient or nonspecific disturbances, the fidgety period likely captures more stable and functionally relevant neurological alterations.^[23]^ The greater S observed in this period suggests that assessments conducted during this time frame provide clinicians with a more reliable basis for early diagnosis and intervention.

Moreover, the methodological advantages of fidgety period assessments – including higher interrater reliability and clearer operational criteria – may further contribute to its superior predictive value.^[24]^ These findings align with previous studies emphasizing the high sensitivity and specificity of fidgety GMs for CP prediction.^[25]^ Importantly, the use of the S in our analysis offers a novel, integrated approach to ranking diagnostic utility by combining both sensitivity and specificity, thus strengthening the confidence in our conclusion.

Nonetheless, the writhing period should not be disregarded. Its clinical utility remains valuable, particularly in settings where early postnatal assessments are prioritized. However, our results suggest that greater emphasis should be placed on fidgety period evaluation in routine neurodevelopmental surveillance protocols.

Despite these strengths, we must comment on the limitations of our analyses. First was the definition of our gold standard, various standards are used as reference standard like saliva culture, urine PCR, and urine culture. Secondly, it is likely that variability existed between institutions with respect to the number of participants. Finally, 4 study was deemed high-risk for bias based on the QUADAS- 2 assessment, the risk of bias was high. All of which cause a considerable heterogeneity.

This meta-analysis has several limitations. First, substantial heterogeneity was observed across the included studies. Although sensitivity analysis identified a single study as the main source of heterogeneity, this variation may still affect the robustness of the pooled estimates. Second, only 8 studies were included in the analysis, which may limit the generalizability and statistical power of the findings. Third, one of the included studies was assessed as having a high risk of bias based on the QUADAS- 2 assessment which may have influenced the overall quality of the evidence and introduced potential bias into the conclusions.

## 5. Conclusion

This meta-analysis shows that the fidgety period of GMs is more valuable than the writhing period in predicting CP. The higher Superiority Index for the fidgety period suggests it is a better indicator for early diagnosis. However, due to the small number of included studies, high heterogeneity, and 1 study with high risk of bias, the results should be interpreted with caution.

## Acknowledgments

AI was used exclusively for editing and refining the language of the article.

Supplemental Digital Content “Supplement files 2-6” are available for this article (https://links.lww.com/MD/P635, https://links.lww.com/MD/P636, https://links.lww.com/MD/P637, https://links.lww.com/MD/P638, https://links.lww.com/MD/P639)

## Author contributions

**Validation:** Taotao Wang, Long Guo.

**Conceptualization:** Bo Zheng.

**Data curation:** Bo Zheng.

**Formal analysis:** Hong Yang, Zhijie Wen, Yuanfei Ye.

**Funding acquisition:** Hong Yang.

**Investigation:** Hong Yang, Hongyan Liu, Yuanfei Ye.

**Methodology:** Hong Yang, Hongyan Liu, Yuanfei Ye.

**Project administration:** Hongyan Liu, Yun Zhou, Yuanfei Ye.

**Resources:** Hongyan Liu, Yun Zhou.

**Software:** Yun Zhou.

**Supervision:** Yun Zhou.

**Visualization:** Taotao Wang, Long Guo.

**Writing – original draft:** Taotao Wang, Long Guo, Bo Zheng, Zhijie Wen.

**Writing – review & editing:** Taotao Wang, Long Guo, Bo Zheng, Zhijie Wen.

## Supplementary Material


