# Supplementary material for: Predictive value and ranking of writhing and fidgety movements for cerebral palsy: A meta-analysis based on the Superiority Index
Source: Medicine (Baltimore). 2025 Aug 15;104(33):e43813. doi: 10.1097/MD.0000000000043813 (PMC12366895; doi:10.1097/MD.0000000000043813)
Supplement: Supplementary file 1 [file medi-104-e43813-s001.pdf]

| Search nu | Query | Sort By | Filters | Search De | Results | Time |
|---|---|---|---|---|---|---|
| 4 | ((((sensitivi | Most Recent | | ("sensitivit | 79 | 22:05:23 |
| 3 | ((sensitivity | Most Recent | | "sensitivity | 1,893,828 | 22:02:33 |
| 2 | (neurologic | Most Recent | | "neurologi | 31,410 | 22:01:53 |
| 1 | general mo | Most Recent | | "general m | 590 | 22:01:34 |
